# Wounding and Phospholipase C Inhibition: Evaluation of the Alkaloid Profiling in Opium Poppy

**DOI:** 10.3390/plants14101413

**Published:** 2025-05-08

**Authors:** Barbora Hans, Ema Balažová, Svetlana Dokupilová, Peter Mikuš, Andrea Balažová, Renáta Kubíková, Marek Obložinský

**Affiliations:** 1 Department of Cell and Molecular Biology of Drugs, Faculty of Pharmacy, Comenius University Bratislava, Kalinčiakova 8, 832 32 Bratislava, Slovakia; barbora.hans@uniba.sk (B.H.); ema.balazova@fpharm.uniba.sk (E.B.); balazova@fpharm.uniba.sk (A.B.); oblozinsky@fpharm.uniba.sk (M.O.); 2 Department of Pharmaceutical Analysis and Nuclear Pharmacy, Faculty of Pharmacy, Comenius University Bratislava, Odbojárov 10, 832 32 Bratislava, Slovakia; dokupilova@fpharm.uniba.sk (S.D.); mikus@fpharm.uniba.sk (P.M.); 3 Toxicologic and Antidoping Centre, Faculty of Pharmacy, Comenius University Bratislava, Odbojárov 10, 832 32 Bratislava, Slovakia

**Keywords:** opium poppy, wounding, benzylisoquinoline alkaloids, secondary metabolites, phospholipase C, methyl jasmonate, transcription factors, high-performance liquid chromatography–mass spectrometry

## Abstract

Wounding triggers complex secondary metabolic pathways in plants, including benzylisoquinoline alkaloid (BIA) biosynthesis in opium poppy (*Papaver somniferum* L.). This study explores transcriptional and metabolic responses to wounding and methyl jasmonate (MeJA) treatment, focusing on BIA biosynthesis and regulatory mechanisms. Real-time expression analysis revealed significant up-regulation of transcripts in the (*S*)-reticuline and papaverine biosynthetic pathway, while the noscapine pathway was suppressed. The morphinan pathway also showed transcriptional activation, except in the case of codeinone reductase (*COR*), which remained unresponsive to both wounding and MeJA, suggesting a partially uncoupled mechanism. Metabolite profiling using HPLC-MS demonstrated a rapid accumulation of morphine post wounding, further supporting the hypothesis of independent regulatory control over *COR*. The role of phospholipase C (PLC) in modulating wound-induced BIA accumulation was investigated, revealing that PLC inhibition reduced morphine production and suppressed *COR* expression. These findings highlight the importance of phospholipid-dependent signalling in activating morphine biosynthesis, potentially at the expense of other BIAs. This study provides insights into plant stress responses and suggests strategies for enhancing BIA production through targeted interventions, offering potential applications in improving alkaloid yield.

## 1. Introduction

Plants produce a diverse array of low-molecular-weight chemical compounds as a defence mechanism against biotic and abiotic stresses [1]. These compounds fall into three major primary classes: alkaloids, terpenoids, and phenolic compounds [2]. Among these, benzylisoquinoline alkaloids (BIAs) have been extensively studied at the molecular level [3]. BIAs are a diverse class of nitrogen-containing plant secondary metabolites found in several plant families, particularly Papaveraceae [4]. With over 2500 known structures [5], BIAs play a crucial role in plant defence against biotic and abiotic stresses [1]. Opium poppy (*Papaver somniferum* L.) is the exclusive commercial source of valuable BIAs, such as morphine, codeine, papaverine [6], and noscapine [7], which have significant pharmaceutical applications [8,9,10].

**Figure 1:**
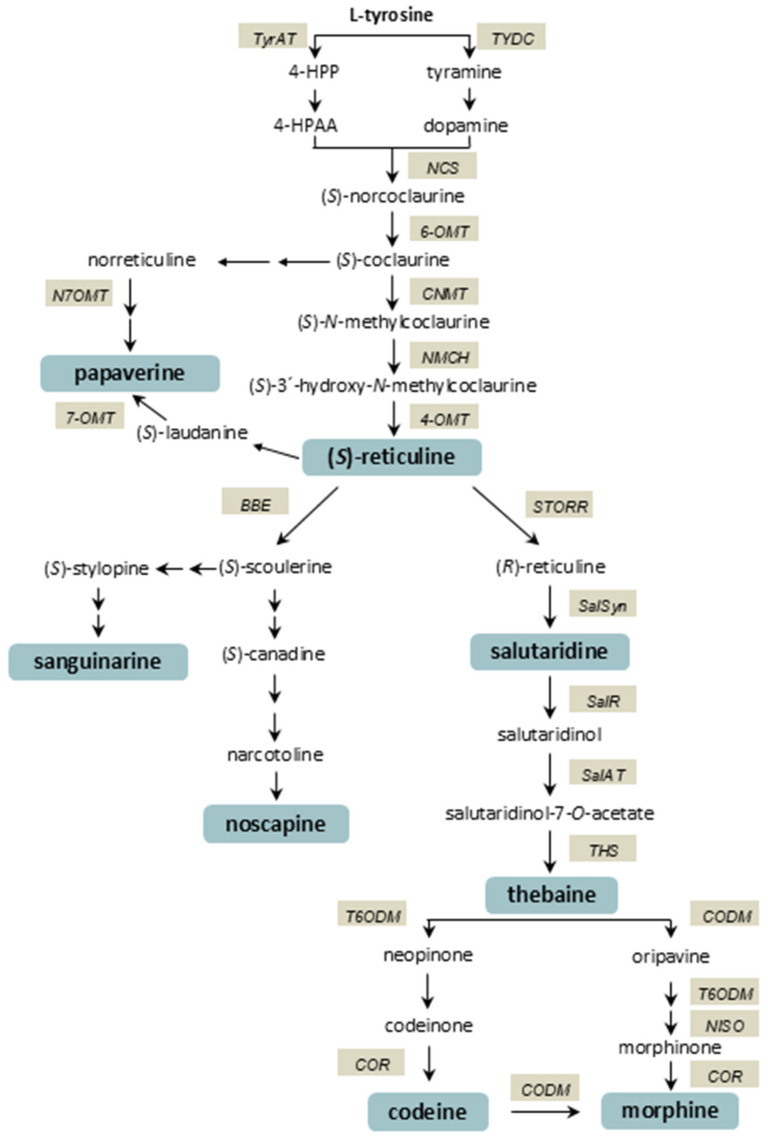
Biosynthetic pathway of benzylisoquinoline alkaloids in opium poppy (*Papaver somniferum* L.). Tyrosine/DOPA decarboxylase (TYDC); tyrosine aminotransferase (TyrAT); 4-hydroxyphenylpyruvate (4-HPP); 4-hydroxyphenylacetaldehyde (4-HPAA); norcoclaurine synthase (NCS); norcoclaurine 6-*O*-methyltransferase (6-OMT); (*R*,*S*)-coclaurine *N*-methyltransferase (CNMT); (*S*)-*N*-methylcoclaurine 3-hydroxylase (NMCH); (*R*,*S*)-3′-hydroxy-*N*-methylcoclaurine 4′-*O*-methyltransferase (4-OMT); (*R*,*S*)-norreticuline 7-*O*-methyltransferase (N7OMT); (*R*,*S*)-reticuline 7-*O*-methyltransferase (7-OMT); berberine bridge enzyme (BBE); STORR fusion protein (STORR); salutaridine synthase (SalSyn); salutaridine reductase (SalR); salutaridinol 7-*O*-acetyltransferase (SalAT); thebaine synthase (THS); thebaine 6-*O*-demethylase (T6ODM); codeinone reductase (COR); codeine *O*-demethylase (CODM); neopinone isomerase (NISO).

The biosynthesis of BIAs in opium poppy follows a complex, highly branched pathway (Figure 1) that begins with the amino acid L-tyrosine [11]. In the early stages of the biosynthetic pathway, tyrosine decarboxylase (TYDC) and norcoclaurine synthase (NCS) catalyse the formation of (*S*)-norcoclaurine, the first committed intermediate in BIA biosynthesis. Through a series of methylation and hydroxylation reactions catalysed by enzymes including norcoclaurine 6-*O*-methyltransferase (6-OMT), coclaurine *N*-methyltransferase (CNMT), and 4′-*O*-methyltransferase (4-OMT), the pathway leads to the formation of (*S*)-reticuline, a crucial branch-point intermediate. From (*S*)-reticuline, various end products are formed via different branches, including the noscapine, papaverine, and morphinan pathways. The initial step in the formation of the phthalideisoquinoline alkaloid noscapine is the conversion of (*S*)-reticuline to (*S*)-scoulerine by berberine bridge enzyme (BBE) [12]. (*S*)-scoulerine is converted to noscapine via eleven steps [13]. (*S*)-reticuline can be converted to papaverine in several steps involving (*R*,*S*)-reticuline 7-*O*-methyltransferase (N7OMT), forming the papaverine branch. The morphinan branch pathway begins with the conversion of (*S*)-reticuline to (*R*)-reticuline by an enzyme known as STORR (S-to-R reticuline) [14,15]. This is followed by the formation of salutaridine by salutaridine synthase (SalSyn). Reduction of the salutaridine is catalysed by salutaridine reductase (SalR), yielding salutaridinol. Salutaridinol is then acetylated on the resulting hydroxyl moiety by salutaridinol 7-*O*-acetyltransferase (SalAT) to produce the unstable intermediate salutaridinol 7-*O*-acetate, which was long thought to spontaneously convert to thebaine. However, it has recently been shown that the final step in thebaine biosynthesis is catalysed by thebaine synthase (THS) [16]. Thebaine is converted to morphine by a multistep bifurcated pathway. The major route involves the conversion of thebaine through several intermediates, including codeine. Codeinone reductase (COR) catalyses the reduction of codeinone to codeine. Finally, morphine is formed by codeine *O*-demethylase (CODM). The isolation of oripavine in an opium poppy led to the discovery of a second, minor route to morphine that bypasses codeinone and proceeds via morphinone [17,18]. Morphine and, to a lesser extent, codeine are the major products of the pathway in plants [19].

BIA biosynthesis is regulated by multiple mechanisms, including hormonal signalling and transcriptional regulation [20]. Several transcription factor families, such as WRKY, MYB, C3H, NAC, and AP2-ERF, seem to play crucial roles in regulating BIA biosynthesis, and up-regulation of the transcription factor is speculated to result in the synchronised accumulation of BIAs in opium poppy [21]. Promoter analyses of BIA-related genes have revealed the presence of WRKY and MYB binding motifs, suggesting their co-regulation [10]. These transcription factors control the expression of multiple biosynthetic genes and are often inducible by wounding and methyl jasmonate (MeJA) treatment [22,23]. Comparative studies in poppy cultivars with contrasting alkaloid profiles have also highlighted the role of TFs in modulating the biosynthesis of specific alkaloids [2,24].

Phospholipid-based signalling has emerged as another layer of regulation in plant stress responses and secondary metabolism. Phospholipases, such as phospholipase A_2_ (PLA_2_) and phospholipase D (PLD), have been implicated in modulating BIA biosynthetic genes and alkaloid accumulation in opium poppy [25]. While the precise activation mechanism of BIA biosynthesis remains elusive, phospholipases are thought to play a pivotal role by generating signalling molecules through the degradation products of membrane phospholipid hydrolysis. Among these, phospholipase C (PLC) is particularly significant as it catalyses the hydrolysis of phosphatidylinositol bisphosphate (PIP_2_) into inositol 1,4,5-trisphosphate (IP_3_) and diacylglycerol (DAG) [26]. IP_3_ is known to increase intracellular calcium (Ca^2+^) concentrations [27], which can activate various kinases, including mitogen-activated protein kinases (MAPKs) [28]. Notably, in soybean, a direct link between phosphoinositide-specific phospholipase C (PI-PLC) activity and MAPK activation has been demonstrated, where PI-PLC influence a MAPK signalling cascade under abiotic stress conditions [26,29]. For instance, in some plants, MAPK pathways have been linked to the regulation of transcription factors such as WRKY [30], which are involved in alkaloid biosynthesis [10]. This interaction could be particularly important in the context of stress responses, where BIA production plays a role in plant defence mechanisms. However, the role of phospholipase C (PLC) and its signalling cascade in secondary metabolism in opium poppy remains largely unexplored.

Additionally, in *Arabidopsis* leaves, a five-fold increase in IP_3_ levels generated by PI-PLC was detected 30 min post wounding. Simultaneously, the precursor lipids transiently decreased, and subsequently, jasmonic acid (JA), a key mediator of wound signalling, accumulated during the first hours post wounding. In JA biosynthesis-deficient mutants, no IP3 accumulation was observed, indicating that IP3 accumulation depends on JA and highlighting its role in early wound signalling [31,32,33].

In opium poppy, many BIA biosynthetic genes are wound-inducible, and inhibition of phospholipases affects alkaloid levels in a wound-dependent manner [22,25]. To elucidate the specific role of PLC, we analysed time-dependent changes in BIA gene expression and alkaloid profiles in wounded opium poppy plants treated with the PLC inhibitor edelfosine [34,35,36], alongside changes induced by MeJA treatment, a well-characterised jasmonate signalling molecule.

## 2. Results and Discussion

To investigate the role of PLC in defence mechanisms triggered by wounding in the leaf tissue of six-week-old opium poppy (*Papaver somniferum* L.) plants treated with PLC inhibitor, real-time expression analysis of transcripts involved in BIA biosynthesis and selected transcription factors was performed at different time points after injury. A control experiment with untreated poppy plants wounded in the same manner was performed for comparison. Metabolite profiling was performed using HPLC-MS in both intact and wounded leaves to link gene expression and changes in alkaloid content. Precursors of poppy alkaloids, such as reticuline and salutaridine, and major alkaloids in the morphinan branch, such as thebaine, oripavine, morphine, and codeine, were analysed. Although BIAs accumulate after seed imbibition [37], in our experiment, noscapine and papaverine were not detectable in all samples, limiting their quantification.

### 2.1. The Impact of Wounding on BIA Biosynthetic Pathway Gene Expression and Alkaloid Content

#### 2.1.1. The S-Reticuline Pathway

**Figure 2:**
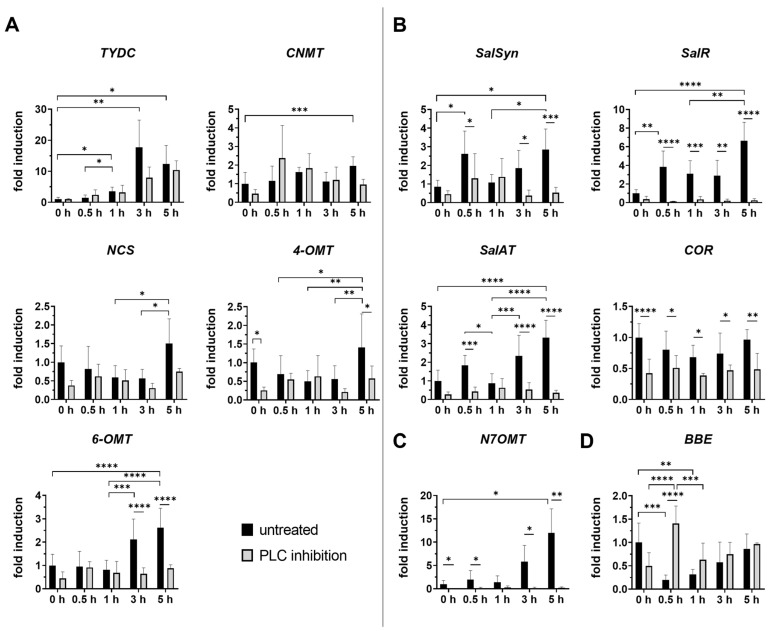
Temporal changes in relative gene expression of selected enzymes of BIA pathway following PLC inhibition in opium poppy (*Papaver somniferum* L.). The figure is divided into four groups, each representing a different branch of the BIA pathway. (**A**) (*S*)-reticuline pathway; (**B**) morphinan pathway; (**C**) papaverine pathway; (**D**) noscapine/sanguinarine pathway. Bar graphs represent fold changes normalised to the untreated and unwounded control at the time point set as 0 h (set to 1). The x-axis represents wounded plants, both untreated (black bars) and treated with PLC inhibitor (grey bars), collected at different time points (0.5 h, 1 h, 3 h and 5 h). Unwounded plants treated with PLC inhibitor (0 h) are shown for comparison to the untreated and unwounded control (0 h) to determine its individual effect on BIA biosynthesis. *β-actin* and *EF1α* were used as reference genes. Selected statistically significant differences are indicated by * (* *p* < 0.05, ** *p* < 0.01, *** *p* < 0.001, **** *p* < 0.0001).

**Figure 3:**
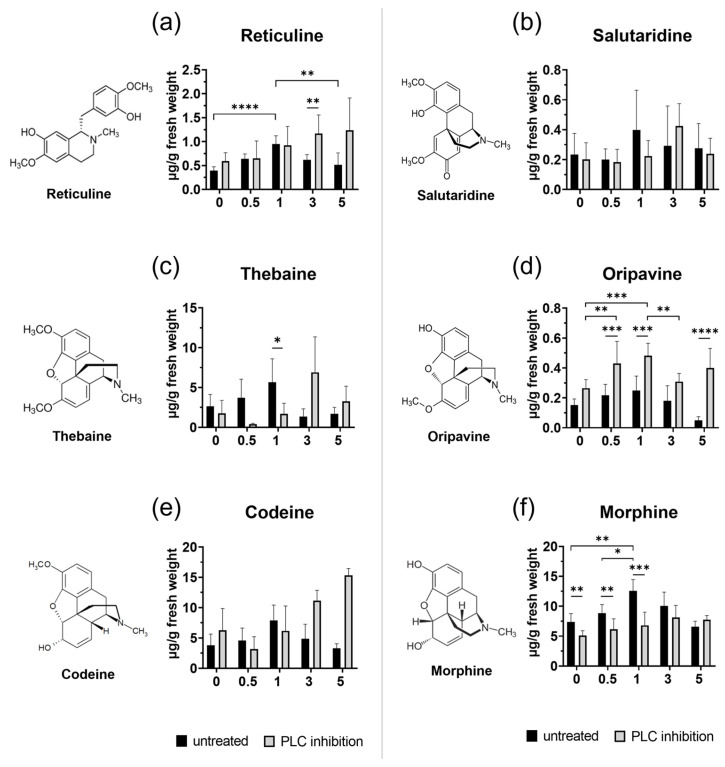
HPLC-MS analysis of benzylisoquinoline alkaloids. Changes in the formation of opium poppy alkaloids in untreated and inhibitor-treated opium poppy (*Papaver somniferum* L.) plants. Bar graphs show calculation in μg alkaloid/g fresh weight (y-axis) at different time points (0 h, 0.5 h, 1 h, 3 h, and 5 h) displayed on the x-axis. Dextromethorphan was used as an internal standard. Selected statistically significant differences are indicated by * (* *p* < 0.05, ** *p* < 0.01, *** *p* < 0.001, **** *p* < 0.0001).

(*S*)-reticuline serves as a key intermediate for the production of various alkaloids, including morphine, codeine, and noscapine. Our data indicate that all examined transcripts (*TYDC*, *NCS*, *6-OMT*, *CNMT,* and *4-OMT*) involved in the (*S*)-reticuline pathway showed significantly increased expression levels at 5 h after injury in untreated plants (Figure 2A). Inhibition of PLC seemed to affect almost all genes after treatment, even in unwounded plants, except *TYDC* copying the trend of transcript in untreated wounded plants. Transcript of *6-OMT* was repressed significantly in all wounded plants with inhibited PLC compared to untreated wounded plants. Interestingly, a significant decrease in *4-OMT* transcript levels was determined 5 h post wounding in PLC-inhibited plants, but simultaneously, the total reticuline content was elevated (Figure 3a), suggesting the accumulation of this intermediate metabolite compared to untreated groups.

Additionally, no substantial changes were observed as an immediate response, suggesting that in physiological conditions, the plant initially focuses on the biosynthesis of downstream precursors or other metabolites crucial for the early stress response. Total reticuline (*S* + *R*) reached its highest level 1 h after wounding in untreated plants (Figure 3a), which seems to be independent of *4-OMT* expression. Similarly, a study by Jablonická et al. (2018) also demonstrated an increase in reticuline levels without a corresponding rise in *4-OMT* in wounded tissue [25].

#### 2.1.2. The Noscapine and Sanguinarine Branch Pathway

In poppy leaf tissue, the transcript for *BBE*, a key enzyme in noscapine and sanguinarine biosynthesis, was clearly down-regulated as an early response to wounding (0.5 h) in untreated plants (Figure 2D). Furthermore, *BBE* transcription did not reach the levels observed in intact untreated plants in any of the wounded poppy groups, neither untreated, nor with inhibited PLC. This finding is consistent with the significant decrease in noscapine content observed by Jablonická et al. (2018) in five-week-old poppy plants post wounding [25], similar to those used in our study. Interestingly, in plants with inhibited PLC, a different trend in *BBE* transcript was observed 30 min post wounding. *BBE* transcript levels increased significantly compared to unwounded plants with inhibited PLC and untreated plants 30 min post wounding. The observed shift in transcription level indicates that PLC plays an important role in the early response to wounding through the regulation of *BBE* transcript. A similar pattern of *BBE* down-regulation was reported in *Arabidopsis thaliana*, where *AtBBE4* expression decreased in response to abiotic stimuli, but increased during pathogen infection [22,38,39]. Mishra et al. (2013) reported that the transcript levels of neither *cheilanthifoline* (*CFS*) nor *stylopine synthase* (*STSY*), following enzymes involved in the biosynthesis of sanguinarine, exhibited any significant changes after wounding in *Papaver somniferum* [22]. These findings suggest that both noscapine and sanguinarine branches may be suppressed after wounding, allowing the plant to prioritise resources for morphinan alkaloid biosynthesis, particularly morphine. Notably, it is known that morphine is metabolised to bismorphine in wounded tissue immediately after injury, facilitating cell wall repair [40]. Furthermore, the regulation of the noscapine and sanguinarine branch pathways may exhibit both temporal and spatial control mechanisms, ensuring that these complex molecules are produced at specific times and, in particular, tissues within the plant.

#### 2.1.3. The Papaverine Pathway

According to Pathak et al. (2013), (*S*)-coclaurine seems to be a primary branchpoint intermediate and preferred route for papaverine biosynthesis, rather than (*S*)-reticuline [24]. Therefore, our research focused on the expression of *N7OMT*, involved in the (*S*)-coclaurine pathway. Our results showed that *N7OMT* transcription was induced 3–5 h after wounding in untreated plants (Figure 2C). On the contrary, inhibition of PLC and subsequent wounding led to significant downregulation of *N7OMT* compared to untreated wounded plants. This difference is the most markable among all analysed transcripts, highlighting a regulatory connection between PLC activity and papaverine biosynthesis. Papaverine is difficult to detect in certain plants that are only a few weeks old. Even in our study papaverine was not detectable in intact plants as well as after wounding. However, Mishra et al. (2013) reported an increase in papaverine concentration 5 h post wounding in leaves of 130-day opium poppy plant compared to unwounded tissue [22], which supports our data, showing an up-regulation of *N7OMT* transcript 5 h after wounding only in untreated plants.

#### 2.1.4. The Morphinan Pathway

In contrast to *BBE*, *SalSyn* expression showed a trend towards up-regulation as an early response to wounding (Figure 2B), mirroring the patterns observed for salutaridine. The wound-induced up-regulation of both *SalSyn* transcript and salutaridine levels suggests that the plant is actively increasing morphinan alkaloid production as part of its defence response to injury. *SalR* and *SalAT*, which share the same branch pathway, also exhibited similar expression patterns after wounding (Figure 2B). This may be due to the existence of a potential metabolic complex involving SalR and SalAT, initially suggested by the unexpected accumulation of salutaridine rather than salutaridinol in *SalAT*-silenced opium poppy plants [41,42]. The coordinated expression of these genes suggests that they may be controlled by one or more regulatory factors.

It is hypothesised that wounding activates the minor pathways leading to morphine biosynthesis, involving the synthesis of oripavine [25]. In our study, we detected wounding-induced changes in both minor (oripavine) and major (codeine) pathways (Figure 3). The level of oripavine fluctuated dynamically at certain time points, while codeine levels increased 2 h after injury.

The observation that *COR* transcript levels remained almost unchanged in wounded tissues (Figure 2B), is consistent with the earlier findings that COR is not induced in response to either elicitor treatment or wounding [22,37], suggesting its tightly regulated role in morphinan alkaloid biosynthesis. Although *COR* expression did not change significantly, the level of morphine increased significantly, peaking at 1 h and gradually declining by 5 h after injury. The consistent lack of *COR* induction after wounding may suggest that post-translational modifications, rather than transcriptional induction, may cause the observed changes under stress conditions.

**Figure 4:**
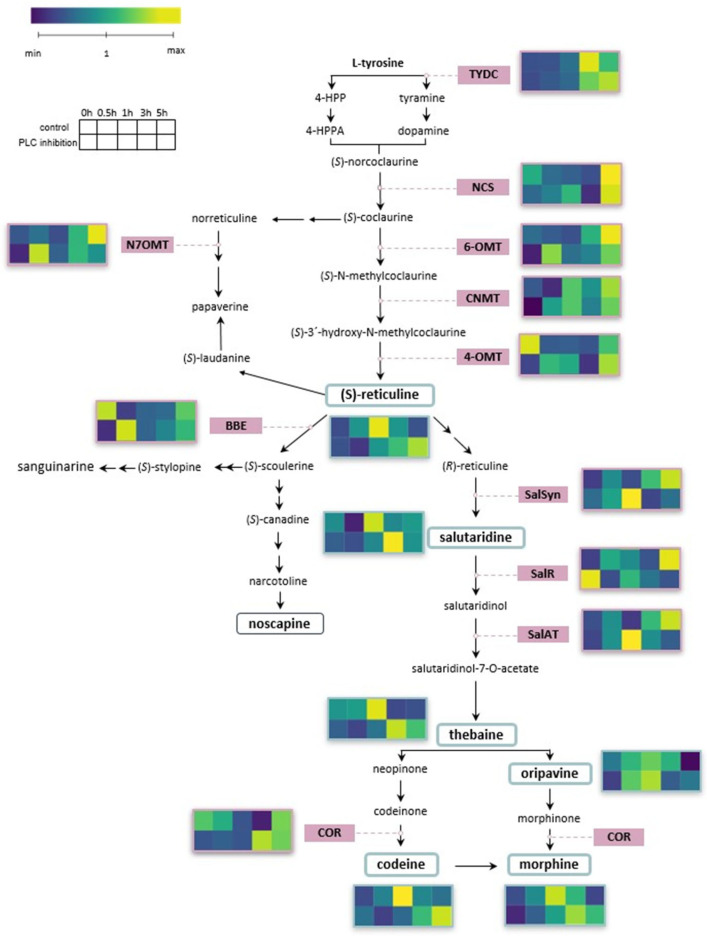
The figure illustrates the biosynthetic pathway of benzylisoquinoline alkaloids and shows heatmaps of up- and down-regulation of both alkaloids and biosynthetic genes in response to wounding. Plants without inhibitor are compared to those with inhibitor at time points 0 h, 0.5 h, 1 h, 3 h, and 5 h post wounding. Values were normalised relative to the unwounded control. The heatmaps in this figure were created using BioRender (https://app.biorender.com/).

The up-regulation of BIA biosynthetic pathway transcripts and the concomitant increase in alkaloid accumulation aligns with the notion that wounding activates both transcriptional and metabolic levels of the BIA pathway (Figure 4 for overview).

### 2.2. Inhibition of PLC Down-Regulates BIA-Related Genes and Drives Changes in Alkaloid Content

Previous research on opium poppy has shown that inhibition of plant phospholipases, specifically phospholipase A_2_ (PLA_2_) and phospholipase D (PLD), affects the accumulation of alkaloids and their corresponding BIA transcripts [25]. Interestingly, these phospholipases appear to have opposing effects and influence different levels of BIA biosynthetic branches. However, the role of phospholipase C (PLC) in this context remained unclear. Given this gap in our understanding, we investigated whether PLC plays a role in BIA metabolism using a selective PLC inhibitor, edelfosine, a synthetic analogue of lysophosphatidylcholine. Edelfosine was chosen for its water solubility, which simplifies application and eliminates the need for solvent control [34], allowing us to focus exclusively on PLC effects.

**Figure 5:**
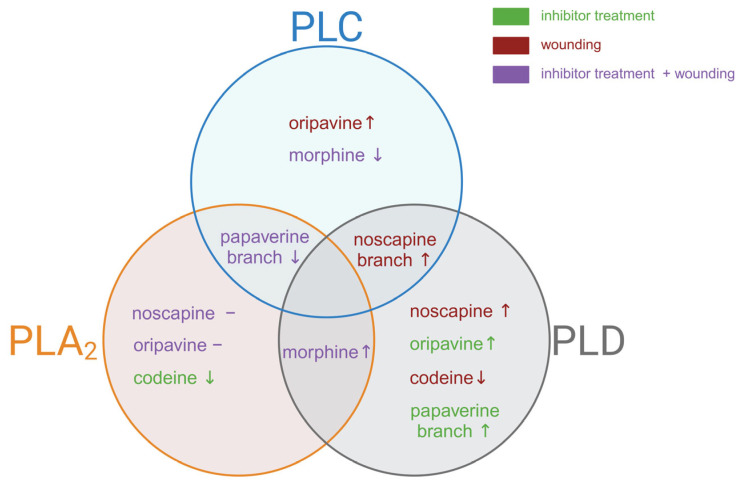
The diagram illustrates the distinct and overlapping effects of inhibition of phospholipases (PLC, PLA_2_, and PLD) on the alkaloid biosynthesis pathway and corresponding gene in opium poppy. The symbols “↑” and “↓” represent an increase and decrease, respectively, and the symbol “–” represents no changes in alkaloid levels or corresponding biosynthetic branch.

Our investigation revealed notable differences in the effects of PLC inhibition across various BIA pathways (Figure 5 for overview). Upon application of edelfosine, we noted a significant decrease in both morphine levels (Figure 3f) and the corresponding *COR* transcript (Figure 2B) (without additional wound treatment), suggesting a direct link between PLC signalling and morphine biosynthesis at the basal level. Interestingly, codeine, which requires the same enzyme as morphine for its conversion from corresponding precursors, showed no substantial decline after treatment with an inhibitor (Figure 3e). In contrast, oripavine levels increased in wounded tissues of the treated plant (Figure 3d), suggesting that this rise might occur at the expense of morphine. Conversely, thebaine content diminished significantly following wounding and inhibition treatment (Figure 3c). Our findings align with previous observations that COR is crucial for thebaine biosynthesis, where its up-regulation increases and down-regulation decreases thebaine content [43]. Taken together, these findings suggest that PLC influences multiple points within the morphinan branch, resulting in differential accumulation of products within this pathway. This complex regulatory pattern is similar to observations made with PLA_2_ inhibition, which led to a decrease in thebaine and codeine content, while simultaneously increasing morphine production at the basal level [25].

Noscapine is synthesised by a specific branch of BIA biosynthesis. As previously mentioned, *BBE* expression decreased in the early stages after wounding. However, when an inhibitor was applied, this down-regulatory effect was reversed (Figure 2D). A study by Jablonická et al. (2018) indicated that phospholipase D acts as a negative regulator of this branch, as inhibition of PLD increased noscapine accumulation and *BBE* expression after wounding [25]. In contrast, PLA_2_ does not appear to regulate this pathway [25]. The observed activation of the branch leading to noscapine and sanguinarine production suggests that both phospholipase C and D may play a sophisticated role in the regulation of plant secondary metabolism.

Another distinct branch of BIA biosynthesis leads to papaverine production. Although the papaverine analysis was challenging, we observed a striking and significant decrease in *N7OMT* expression across all inhibited samples (Figure 2C). This down-regulation was the most pronounced among all analysed transcripts, suggesting a strong regulatory link between phospholipase C activity and papaverine biosynthesis. Similarly, phospholipase A_2_ seems to act as a positive regulator, whereas phospholipase D seems to act as a negative regulator of this branch [25].

### 2.3. Response of BIA-Related Genes to Exogenous Application of MeJA

**Figure 6:**
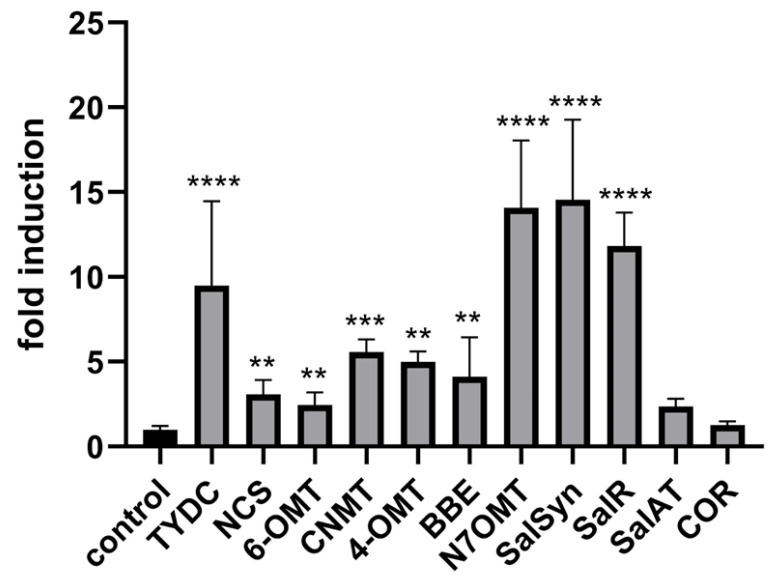
Changes in relative gene expression of selected enzymes of BIA pathway following MeJA treatment in opium poppy (*Papaver somniferum* L.). Bar graphs represent fold changes normalised to the untreated control (set to 1). *β-actin* and *EF1α* were used as reference genes. Statistically significant differences are indicated by * (** *p* < 0.01, *** *p* < 0.001, **** *p* < 0.0001).

Previous studies have shown that MeJA treatment triggers a cascade of responses in opium poppy leading to increased alkaloid accumulation [44,45,46]. This process involves the activation of jasmonate-responsive genes, which play a crucial role in plant defence mechanisms and secondary metabolite production. In our study, exogenous application of methyl jasmonate to intact plants significantly induced BIA-related genes including *TYDC*, *CNMT*, *NCS*, *6-OMT*, *4-OMT*, *SalSyn*, *SalR*, *SalAT*, *N7OMT*, and *BBE* (Figure 6) However, the *COR* transcript was not significantly induced in intact plants following MeJA application (Figure 6). Notably, *COR* was the only transcript in our study that was not induced by either wounding or methyl jasmonate under the same conditions. This finding aligns with [45], who observed that *COR* did not increase in response to MeJA treatment in opium poppy cell cultures and remained at a relatively constant level throughout the 24 h period studied, unlike other BIA genes examined. The differential induction of BIA-related genes, except *COR*, further supports the notion that there is at least a partially uncoupled mechanism for their activation.

### 2.4. Transcriptional Regulation of BIA Pathway

**Figure 7:**
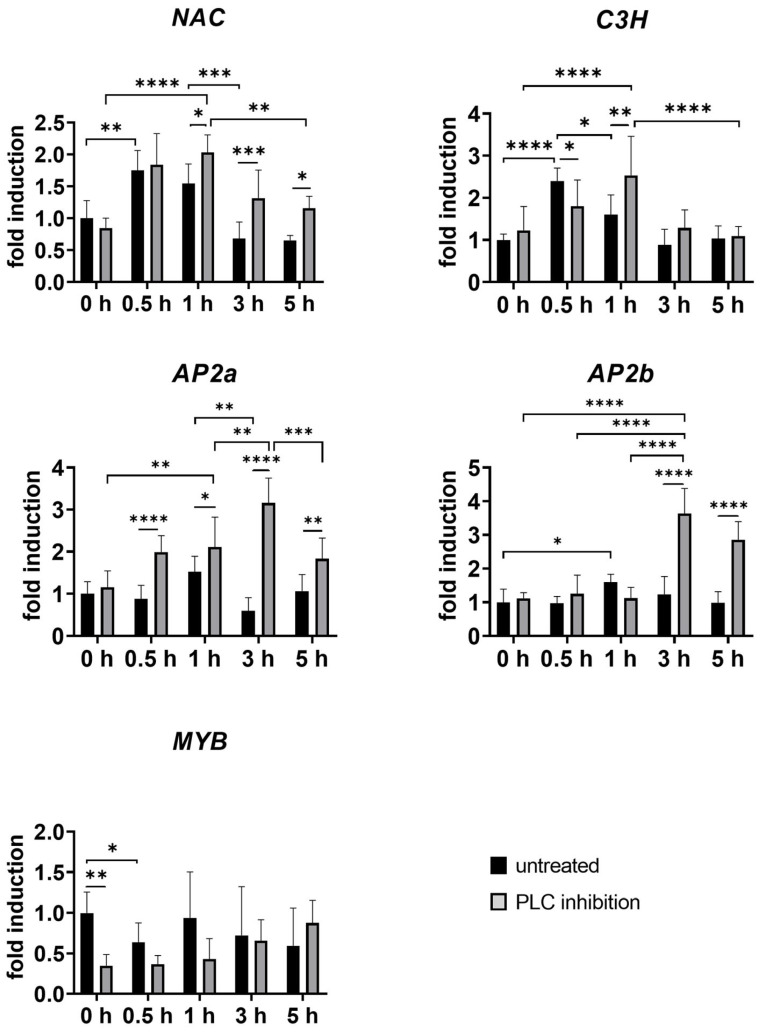
Temporal changes in relative gene expression of selected TFs following PLC inhibition in opium poppy (*Papaver somniferum* L.). Bar graphs represent fold changes normalised to the untreated and unwounded control at the time point set as 0 h (set to 1). The x-axis represents wounded plants, both untreated (black bars) and treated with PLC inhibitor (grey bars), collected at different time points (0.5 h, 1 h, 3 h, and 5 h). Unwounded plants treated with PLC inhibitor (0 h) are shown for comparison to the untreated and unwounded control (0 h). *β-actin* and *EF1α* were used as reference genes. Selected statistically significant differences are indicated by * (* *p* < 0.05, ** *p* < 0.01, *** *p* < 0.001, **** *p* < 0.0001).

In the context of BIA biosynthesis, MYB TF family plays a significant role. Notably, *MYB* expression was substantially reduced after wounding (Figure 7), highlighting its potential involvement in the early response to injury. This reduction was further observed in unwounded tissues after inhibitor treatment (Figure 7), indicating that PLC also influences the MYB TF family. According to Vergnolle et al. (2005), inhibition PLC activity also blocked cold-induced *MYB73* expression in *A. thaliana*, suggesting that PLC activity is upstream from the activation of *MYB73* [34]. MYB family genes are also implicated in noscapine biosynthesis, with their elements present in the promoter regions of genes involved in this process, and are shown to be co-expressed [10,20,47]. In silico analysis revealed that MYB TF members are the most abundant in noscapine-rich opium poppy cultivars, suggesting a specific role in BIA biosynthesis [48]. The observed effects of PLC inhibition on *MYB* transcription factor and *BBE* levels suggest a broader regulatory role of PLC in modulating the expression of transcription factors and biosynthetic enzymes. Notably, our findings support the hypothesis that phospholipase C inhibition impacts transcription factor expression.

Recent studies have highlighted the significant role of the APETALA2/ethylene responsive factor (AP2/ERF) in regulating alkaloid biosynthesis across various plant species. For instance, it has been suggested that the AP2/ERF TF regulates the expression of *6-OMT* in California poppy [3]. Our study examined the expression of two members of the AP2/ERF TF family: *PsAP2*, first isolated by Mishra et al. (2015) [23], and a second member annotated as *AP2b* in this study. Consistent with previous findings for *PsAP2* by Mishra et al. (2015) [23], both AP2 members showed up-regulation at 1 h post wounding, with expression gradually decreasing after 3 h and 5 h, respectively (Figure 7). After inhibition, both TFs were highly up-regulated after wounding compared to untreated controls, suggesting their similar role in stress responses. Recent research has also revealed that AP2/ERF can interact with other TF families, such as MYB, to regulate secondary metabolite biosynthesis [49].

In our study, transcription factor *Ps*175C3H, a member of the C3H family, showed a significant induction just 30 min post wounding (Figure 7). This makes C3H the most wound-responsive TF in our study, showing rapid induction after tissue damage and suggesting a pivotal role in the early wound-response cascade. Previous research has implicated *Ps*175C3H in thebaine and oripavine biosynthesis in poppy cultivars, as *Ps*175C3H was not expressed in the Przemko cultivar [2], considered an oripavine- and thebaine-less cultivar [50]. The presence of Zn-finger motifs in *Ps*175C3H, similar to those regulating monoterpenoid indole alkaloid biosynthesis in *Catharantus roseus* [51], suggests a similar regulatory function in *Papaver somniferum* L. [2]. Interestingly, after phospholipase C inhibition, thebaine and oripavine were differentially regulated, indicating complex interactions between transcription factors, signalling pathways, and alkaloid production in response to wounding.

Similarly, the NAC transcription factor showed a significant increase as an early response to wounding, followed by a substantial decrease 3 h after wounding (Figure 7). While the role of NAC in the stress response and secondary metabolite biosynthetic pathway is less studied [52], several NAC TFs are known to be up-regulated in response to oxidative stress, which is crucial for elicitor-induced alkaloid biosynthesis [53,54]. The similar expression patterns of *C3H* and *NAC* suggest their involvement in a regulatory network associated with the initial stress response, potentially triggering downstream stress-responsive genes and secondary metabolite biosynthetic pathways.

Among transcription factors, the WRKY family appears to be the most extensively studied in relation to BIA biosynthesis. These plant-specific TFs regulate gene expression by binding to W-box elements in target promoters [55]. In our study, we examined the expression patterns of two putative WRKY family members. They are candidate regulators of papaverine biosynthesis, particularly in opium poppy, according to Agarwal et al. (2016) [2]. However, they were not conclusively identified as true transcription factors. Interestingly, their expression showed contrasting responses: wounding (without inhibitor treatment) and phospholipase C inhibition had opposite effects on their expression levels. This divergent response suggests the potential for differential regulation of the biosynthetic pathway under varying conditions.

All examined transcription factors and BIA genes (except for *COR*) were wound-responsive. Given that wounding is known to trigger stress responses in plants, including the production of secondary metabolites, this suggests that the TFs we studied may potentially act as regulators in BIA biosynthesis.

The exact mechanisms by which phospholipases modulate alkaloid production remain unclear. It is possible that the PI-PLC pathway controls gene expression by increasing its products or by decreasing its substrates [35]. Previous studies have linked phospholipase signalling pathways to the MAPK cascade. It is well documented that MAPK activity increases rapidly after wounding [56]. This connection is mediated by phospholipase-generated signal molecules like phosphatidic acid (PA), produced by both PLC and PLD [57]. However, it remains unclear whether PLC and PLD activate separate pathways or co-activate a single pathway [35]. In addition, MAPKs have been shown to phosphorylate transcription factors [58], providing a potential link between phospholipases and transcription factors in the downstream regulation of secondary metabolite biosynthesis. Clarifying how phospholipase signalling regulates plant defence responses, potentially involving coordinated transcription factor networks for each biosynthetic pathway, may contribute to future genetic engineering strategies to optimise opium poppy cultivation and lead to improved production of medicinally important alkaloids.

## 3. Materials and Methods

### 3.1. Plant Material and Growth Conditions

Opium poppy plants (*Papaver somniferum* L., cultivar Lazur) were cultivated using a hydroponic system. Seeds obtained from the Medicinal Plant Garden of the Pharmaceutical Faculty of Comenius University were surface-sterilised and germinated on moistened perlite within a climate-controlled growth chamber. Dark conditions were maintained at 25 °C within 60–70% relative humidity until the fifth day post imbibition. Five-day-old seedlings were transferred to ½-strength Hoagland’s solution [59]. Growth conditions comprised a 16 h light/8 h dark photoperiod with a light intensity of 7000 lx maintained at 20–22 °C and 60% relative humidity. After two days, the nutrient solution concentration was increased to 1× Hoagland’s solution, and roots were supplied with supplemental aeration. The hydroponic solution was replenished biweekly.

### 3.2. Experimental Treatments and Wounding

After 42 days of hydroponic growth, *P. somniferum* L. were subjected to a specific treatment. Plants were transferred to 200 mL of fresh Hoagland’s solution (control), Hoagland’s solution supplemented with the PLC inhibitor, edelfosine (1-*O*-octadecyl-2-*O*-methyl-rac-glycero-3-phosphorylcholine), or Hoagland’s solution supplemented with MeJA. The inhibitor was obtained from Cayman Chemical (Item No. 60912, Tallinn, Estonia) and liquid MeJA from Sigma-Aldrich (Item No. 392707, St. Louis, MO, USA). According to the manufacturer’s instructions, edelfosine was dissolved in water to prepare a clear solution. The final concentration of edelfosine in Hoagland’s solution was 150 μM [36], and the final concentration of MeJA was 100 μM [21,60]. Following a 24 h incubation period, six plants (biological replicates) from each group (untreated and treated) were harvested, serving as a control for each created category (untreated = control, MeJA, PLC inhibitor). The remaining plants in each category were mechanically wounded using sterilised tweezers to create superficial scratches on the leaves. Immediately after the wounding, plants were incubated for a further 30 min, 1 h, 3 h, and 5 h. Leaf material from six plants in each time point after wounding was collected, immediately flash-frozen in liquid nitrogen, and ground to a fine powder using a FastPrep-24^TM^ 5G Instrument (MP Biomedicals, Solon, OH, USA).

### 3.3. Gene Expression Analysis by Real-Time PCR

Total RNA from poppy leaf tissue was extracted using the TRIzol method [61]. The RNA quantity and quality were assessed using a Agilent BioTek Epoch Microplate Spectrophotometer with Take3 microvolume plate (Agilent Technologies, Winooski, VT, USA). For cDNA synthesis, 1 µg of total RNA was reverse transcribed using a Maxima H Minus First Strand cDNA Synthesis ds-Nase Kit (Thermofisher, Waltham, MA, USA), following the manufacturer’s instructions. Gene expression analysis was performed using a SYBR Green-Based Real-Time PCR Assay on the QuantStudio 3 System (Applied Biosystems, Waltham, MA, USA). Each 20 μL reaction contained 10 μL of 2× Maxima SYBR Green qPCR Master Mix (Thermofisher, Waltham, MA, USA), 1 μM of each forward and reverse primer (final concentration), 1.5 μL of template cDNA, and 4.5 μL of nuclease-free water. The following thermal cycling conditions were employed: initial denaturation at 95 °C (10 min) and 40 cycles at 95 °C (15 s), 60 °C (30 s), and 72 °C (30 s). This was followed by a dissociation gradient: 95 °C (15 s), 60 °C (1 min), 95 °C (15 s), and 60 °C (15 s). Six biological replicates and two technical replicates were performed for each time point after wounding (0.5 h, 1 h, 3 h, and 5 h) in three categories of plants (untreated, MeJA, PLC inhibitor). Unwounded plants served as the control group and are referred to as time 0 h in the figures. Primer sequences used in this study are listed in the Supplementary Material. qPCR data were analysed using LinRegPCR software (version 2021.2) [62] to determine the initial template concentration (NO) for each sample. Relative gene expression was then calculated based on these NO values in subsequent calculations. As a reference, genes *β-actin* and *EF1α* were used. Primers used for real-time PCR are listed in the Supplementary Materials (Table S1).

### 3.4. Alkaloid HPLC-MS Analysis

#### 3.4.1. Alkaloid Extraction

Alkaloids were extracted from *P. somniferum* L. leaf tissues. To a sample containing 50 mg of plant material (accurately weighed) and 300 µL of methanol (50%, *v*/*v*), 20.0 µL of a stock solution of dextromethorphan was added (1 mg/L), which was used as an internal standard. The sample was centrifuged (15,000× *g*, 10 min) and 150 µL of the resulting supernatant was transferred to a clean vial. The collected supernatant was evaporated under a stream of nitrogen and subsequently reconstituted with 150 µL of 5% acetonitrile solution (initial composition of the mobile phase). The prepared sample was used for HPLC-MS analysis (injected volume 5 µL). Six biological replicates and three technical replicates were performed for each time point after wounding (0.5 h, 1 h, 3 h, and 5 h) in three categories of plants (untreated, MeJA, and PLC inhibitor). Unwounded plants served as the control group and are referred to as time 0 h in the figures.

#### 3.4.2. HPLC-MS Analysis

##### Instrumentation

HPLC-MS measurements were carried out using an Agilent analyser (Agilent Technologies, Santa Clara, CA, USA) composed of the following parts: (i) an Agilent 1260 Infinity HPLC pump, (ii) an Agilent 1260 HiPals dosing device, (iii) an Agilent 1290 TCC column thermostat, (iv) an Agilent 6520 Accurate Mass Q-TOF LC/MS mass spectrometer, and (v) a computer with Mass Hunter software (MassHunter Workstation version B.05.01). An Accucore™ aQ C18 Reversed Phase Column (3.0 × 100 mm, 2.6 µm), obtained form Thermo Fisher Scientific, Waltham, MA, USA, was employed as a chromatographic column.

##### Chemicals

Solvents used for the separations included acetonitrile and water (LC-MS grade), from VWR International, Radnor, PA, USA, and formic acid (<98%), from Fluka Chemie GmbH, Buchs, Switzerland.

Dextromethorphan was used as an internal standard for HPLC analyses.

##### HPLC-MS Operating Conditions

HPLC-MS was performed on an Accucore™ aQ C18 Reversed Phase Analytical Column using mobile phases A: 0.1% aqueous formic acid and B: 100% acetonitrile. Gradient elution was used with an initial mobile phase composition of 5% mobile phase B, which was maintained for 1 min. The mobile phase composition was then linearly increased to 95% mobile phase B for 10 min and then maintained at this level for 4 min. The column was reconditioned with the initial mobile phase composition for 6 min. The flow rate during the entire analysis period was 300 µL/min. The analyses were performed at 40 °C.

The following MS parameters were used to determine the alkaloid content in the samples: drying gas temperature—360 °C, drying gas flow rate—12 L/min, collision gas pressure—60 psi, capillary voltage—3500 V, fragmentor voltage—100 V, skimmer voltage—65 V, and collision gas—N_2_. The mass spectrometer, equipped with electrospray ionisation, operated in positive ionisation mode. To assess the content of individual alkaloids, extracted chromatograms were monitored for *m*/*z* ratios [M+H]^+^ of the adducts of individual alkaloids (±10 ppm) according to Table 1. The peak areas of individual alkaloids were corrected (normalised) with respect to the peak area of the internal standard.

### 3.5. Statistic Analysis

All data were visualised using GraphPad Prism version 10.4.1. Data are presented as mean ± standard error of the mean (SEM). Statistical significance was determined using analysis of variance (ANOVA) followed by Tukey’s HSD test for multiple comparisons. Differences were considered statistically significant at *p* < 0.05.

## 4. Conclusions

In summary, our findings demonstrate that the interruption of phospholipid signalling pathways leads to alterations in the secondary metabolism of BIAs in opium poppy. Our research reveals that poppy plants may simultaneously activate one pathway while suppressing another branch of BIA production, each triggered by distinct signalling cascades. Due to the complexity of BIA biosynthesis, identifying specific targets is currently challenging.

## Figures and Tables

**Table 1:** *m*/*z* ratios of [M+H]^+^ adducts of individual alkaloids used for extraction of chromatograms.

Analyte	*m*/*z* for [M+H]^+^	Analyte	*m*/*z* for [M+H]^+^
*Dextromethorphan*	272.2009	*Salutaridine*	328.1543
*Morphine*	286.1448	*Reticuline*	330.1700
*Oripavine*	298.1438	*Papaverine*	340.1530
*Codeine*	300.1594	*Noscapine*	414.1547
*Thebaine*	312.1594		

## Data Availability

The data presented in this study are available on request from the corresponding author.

## Supplementary Materials

The following supporting information can be downloaded at: https://www.mdpi.com/article/10.3390/plants14101413/s1, Table S1: Oligonucleotide sequences used for expression analysis through qRT-PCR.

## Abbreviations

The following abbreviations are used in this manuscript:

4-HPAA	4-hydroxyphenylacetaldehyde
4-HPP	4-hydroxyphenylpyruvate
4-OMT	(*R*,*S*)-3′-hydroxy-*N*-methylcoclaurine 4′-*O*-methyltransferase
6-OMT	norcoclaurine 6-*O*-methyltransferase
7-OMT	(*R*,*S*)-reticuline 7-*O*-methyltransferase
AP2/ERF	APETALA2/ethylene responsive factor
BBE	berberine bridge enzyme
BIA(s)	benzylisoquinoline alkaloid(s)
CFS	cheilanthifoline synthase
CNMT	(*R*,*S*)-coclaurine *N*-methyltransferase
CODM	codeine O-demethylase
COR	codeinone reductase
DAG	diacylglycerol
HPLC-MS	High-performance liquid chromatography-mass spectrometry
IP_3_	inositol 1,4,5-trisphosphate
JA	jasmonic acid
MAPK(s)	mitogen-activated protein kinase(s)
MeJA	methyl jasmonate
N7OMT	(*R*,*S*)-norreticuline 7-*O*-methyltransferase
NAC	NACNAM/ATAF1,2/CUC2 family protein
NCS	norcoclaurine synthase
NISO	neopinone isomerase
NMCH	(*S*)-*N*-methylcoclaurine 3-hydroxylase
PA	phosphatidic acid
PI-PLC	phosphoinositide-specific phospholipase
PIP_2_	phosphatidylinositol bisphosphate
PLA_2_	phospholipase A_2_
PLC	phospholipase C
PLD	phospholipase D
*Ps*175C3H	C3H transcription factor from *Papaver somniferum* L.
*Ps*AP2	APETALA2/ethylene responsive factor from *Papaver somniferum* L.
RT-qPCR	real-time quantitative polymerase chain reaction
SalAT	salutaridinol 7-*O*-acetyltransferase
SalR	salutaridine reductase
SalSyn	salutaridine synthase
STORR	STORR fusion protein
STSY	stylopine synthase
T6ODM	thebaine 6-*O*-demethylase
TF(s)	transcription factor(s)
THS	thebaine synthase
TYDC	tyrosine decarboxylase
TyrAT	tyrosine aminotransferase
