# Supplementary material for: Wounding and Phospholipase C Inhibition: Evaluation of the Alkaloid Profiling in Opium Poppy
Source: Plants (Basel). 2025 May 8;14(10):1413. doi: 10.3390/plants14101413 (PMC12114792; doi:10.3390/plants14101413)
Supplement: Supplementary file 1 [file plants-14-01413-s001.zip › plants-3569344-supplementary.pdf]

Supplementary Table S1: Oligonucleotide sequences used for expression analysis through qRT-PCR .

Gene name	Forward (5′-3′)	Reverse (5′-3′)	Source
WRKYa	CCATTGAGCATCAGATGAATTGTTGTC	TCTCTTTACTTCCAGCTCCTCCACC	[2]
WRKYb	CATACAAGATAACACCACAGCTCCAGA	TTGTGGAAGCCCGAACTATGCTG	[2]
MYB	GCAGTCTGAAGAAGTTAGCAGTGTAG	ACCTCTCTATCTCCTCTGCTTTCC	[2]
C3H175	CCACTGGCACTTGGGTCTCCTTCG	TGGGTGGGTGGCGGCGTT	[2]
NAC	CTACAACGGCAGAGCACCACGGG	TGGCTAAGTTATAGTAGTAAGTTG	-
PsAP2	TGAAGCTGCTGCTGTTGTTGA	TCTCCACTTTGTAAACCTCTGAATATGA	[23]
AP2b	CGGCGTACGCGTATGATGTTGC	CGTAATGCACCAAGTCGCGCTC	[2]
TYDC	AACAAAGCTACGGAGTCAAGACAA	CGGCGACTGAGTGCGATT	[25]
NCS	CAGGCAATGGTGGAGTTGGT	CCGTGGCACTGCACCTAGA	[25]
6-OMT	CAACAATGTCAAACCCATGTCTTT	CGGAACAGACGGTCTTCGTT	[25]
CNMT	CCAACATGGAAGCAACATGAAA	GTCCAACGTTGTTGATTCATCAG	[25]
4-OMT	GTTTAGATGCAAAACCAG	CCTAGTATCATCGGCACC	[25]
N7OMT	GGTCTCAACAATCAATGGTTCC	GCTCTTTCCTAACGCTGTCTC	[25]
BBE	CGGAAACAGCTTGGGTTGA	CGGACACCAACCAGCAGTAA	[25]
SalSyn	GGTTCCAATGATCGTCATAAGCGA	ATATCAGGTACATCTCTAGCGGAATAA	[25]
SalR	ACTGGCGGAAACAAGGGTATCGGAT	AACAGCTTCATGACCTTTAGTTACATCTCT A	[25]
SalAT	TCCGCGAGTGTCCCTAAGG	TTCCACCACAGTCAAACATGTTC	[25]
COR	GAAATAGAAAGAAAAATGGAGAGTAATGG T	TTCAACTGTTCCCATACCTAAAGCA	[25]
β-actin	TCTCAACCCAAAGGCTAATCG	CCCCAGAATCCAAGACAATAC	[25]
EF1α	TGCTCCAGTTCTTGACTGTC	GTCTCTACAACCATGGGCTTG	[25]
